# Excessive Daytime Sleepiness among Hypertensive US-Born Blacks and Foreign-Born Blacks: Analysis of the CAATCH Data

**DOI:** 10.1155/2013/852389

**Published:** 2013-01-28

**Authors:** N. Williams, O. Abo Al Haija, A. Workneh, D. Sarpong, E. Keku, G. Ogedegbe, S. I. McFarlane, G. Jean-Louis

**Affiliations:** ^1^Department of Medicine, Brooklyn Health Disparities Center, SUNY Downstate Medical Center, NY 11203-2098, USA; ^2^Jackson State University Medical Systems, Jackson Heart Study, Jackson, MS 39213, USA; ^3^Department of Public Health and Preventive Medicine, St George's University, Grenada; ^4^Division of Internal Medicine, Center for Healthful Behavior Change, NYU Medical Center, NY 10016, USA; ^5^Division of Endocrinology, Department of Medicine, SUNY Downstate Medical Center, NY 11203-2098, USA; ^6^Department of Medicine, Sleep Disorders Center, SUNY Downstate Medical Center, NY 11203-2098, USA

## Abstract

*Background*. Evidence shows that blacks exhibit greater daytime sleepiness compared with whites, based on the Epworth Sleepiness Scale. In addition, sleep complaints might differ based on individuals' country of origin. However, it is not clear whether individuals' country of origin has any influence on excessive daytime sleepiness (EDS). *Study Objectives*. We tested the hypothesis that US-born blacks would show a greater level of EDS compared with foreign-born blacks. The potential effects of sociodemographic and medical risk were also determined. *Design*. We used the Counseling African-Americans to Control Hypertension (CAATCH) data. CAATCH is a group randomized clinical trial that was conducted among 30 community healthcare centers in New York, yielding baseline data for 1,058 hypertensive black patients. *Results*. Results of univariate logistic regression analysis indicated that US-born blacks were nearly twice as likely as their foreign-born black counterparts to exhibit EDS (OR = 1.87, 95% CI: 1.30–2.68, *P* < 0.001). After adjusting for effects of age, sex, education, employment, body mass index, alcohol consumption, and smoking habit, US-born blacks were 69% more likely than their counterparts to exhibit EDS (OR = 1.69, 95% CI: 1.11–2.57, *P* < 0.01). *Conclusion*. Findings demonstrate the importance of considering individuals' country of origin, in addition to their race and ethnicity, when analyzing epidemiologic sleep data.

## 1. Introduction

There has been an increased interest in the study of the influence of race/ethnicity on sleep in the last decade. This is fueled in part by epidemiologic evidence showing that individuals of the black race/ethnicity are at increased risk for sleep apnea [1]. An important community-based study involving older black and white participants showed that blacks experienced severe sleep apnea with a relative risk twofold as great (relative risk = 2.13) as that of their counterparts [1]. Ethnic disparities are not noted only among older adults. Indeed, in a case-control family study of sleep apnea [2], comparing 225 blacks and 622 whites, ages 2 to 86 years, 31% of blacks versus 10% of whites had respiratory disturbance index greater than 10.

Although blacks are characterized by greater sleep disturbance as observed among patients with sleep apnea, epidemiologic evidence has shown that they are less likely to report sleep problems compared with whites [3]. In a previous paper examining racial/ethnic differences in the rate of sleep complaints (defined as either difficulty initiating sleep, difficulty maintaining sleep, or early morning awakening among older adults), we showed that rates of sleep complaints among white men and women were 41% and 75%, respectively; among black men and women, they were 14% and 37%, respectively [4]. There is also evidence that sleep complaints might differ on the basis of individuals' country of origin. Results of our previous study conducted in Brooklyn, NY, USA have shown that the prevalence of sleep complaints among African Americans was 71%; among English-Speaking Caribbeans, the prevalence was 34%; Haitians, 33%; Dominicans, 73%; Eastern Europeans, 77%; and European Americans, 70% [5].

To date, little is known about the influence of country of origin on excessive daytime sleepiness, an important marker of disturbed sleep. Data from a hospital-based sleep clinic have shown that blacks are characterized by greater daytime sleepiness compared with whites based on the Epworth Sleepiness Scale [6]. Using data from the Counseling African-Americans to Control Hypertension (CAATCH) Trial, we tested the hypothesis that US-born blacks would show a greater level of excessive daytime sleepiness compared with foreign-born blacks. The potential effects of sociodemographic and medical risk were also determined.

## 2. Methods

The Counseling African-Americans to Control Hypertension (CAATCH) Trial is a group randomized clinical trial that was performed among 30 community healthcare centers (CHC). For the purpose of the present analysis, we focused on baseline sociodemographic and clinical data. Details of the CAATCH data acquisition procedures have been published previously [7]. From 2006 to 2009, a total of 1,058 patients (mean age: 46.75 ± 16.23 years) were recruited and studied to achieve project aims.

### 2.1. Procedures

Patients were enrolled if they met the following criteria: self-identification as black or African-American, were at least 18 years old, were receiving care at the participating CHC for at least 6 months period, had a diagnosis of hypertension and uncontrolled blood pressure (BP) at the last office visit (BP ≥140/90), and were taking at least 1 antihypertensive medication. In addition, all patients must have had uncontrolled BP (systolic BP ≥140 mm Hg or diastolic BP ≥90 mm Hg) at the time of the consent visit, as measured by BPTru (VSM Medtech, Model BPM-300), an automated oscillometric validated BP monitor [3, 8]. Patients were excluded if they were non-English speaking, had an arm circumference of >42 cm, participated in other HTN-related trials, used home BP monitoring currently, had cognitive impairment with Mini Mental Status Examination (MMSE) score <24 for patients with >8th grade education, or MMSE <17 for those with an eighth grade education, or were unwilling or unable to complete screening or baseline assessments, or unwilling or unable to sign informed consent [7].

Trained research assistants interviewed the participants to obtain baseline clinical and sociodemographic data used in the present analysis. Excessive daytime sleepiness was assessed using the Epworth Sleepiness Scale [6, 9]. Validation study showed that the questionnaire has a sensitivity of 0.94, specificity of 0.79 (based on a clinical cut-off of AHI >5), positive predictive value of 0.91, and negative predictive value of 0.86 [10]. Patients' sociodemographic variables including age, gender, income, education, and employment were collected using an instrument developed by the Clinical Directors Network (CDN). All enrolled patients provided informed consent under the supervision of the IRB at New York University Medical Center [7].

### 2.2. Statistical Analysis

Frequency and measures of central tendency were used to describe the sample. ANOVA was used for group mean comparisons, and Chi-square test was employed to assess differences in categorical variables. To test the hypothesis that UBB participants had a greater likelihood of exhibiting EDS than FBB participants, we utilized multivariate logistic regression modeling. Covariates entered in the model were age, sex, education, income, history of alcohol consumption, smoking habit, and body mass index. Before constructing the model, univariate logistic regressions were performed to assess associations between hypothesized predictors and the dependent variable, EDS; only predictors showing a *P* value < 0.05 were entered in the final model [11]. All analyses were performed using SPSS 18.0.

## 3. Results

One thousand and fifty-eight participants provided baseline data for the analysis; 73% were US-born blacks (UBB), 27% were foreign-born blacks (FBB). As illustrated in Table 1, there were no significant group differences in terms of age, gender, and body mass index. However, FBB participants were more likely to be employed, but less likely to have received more than a high school education, less likely to report alcohol consumption, and less likely to report a smoking history.

**Figure 1:**
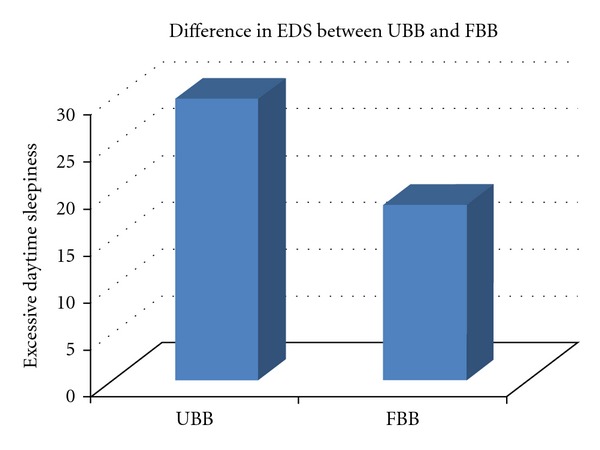
Illustration of difference in excessive daytime sleepiness between US-born blacks and foreign-born blacks (*χ* ^2^ = 12.61, *P* < 0.001).

Results of univariate logistic regression analysis indicated that UBB participants were nearly twice as likely as their FBB counterparts to exhibit EDS (OR = 1.87, 95% CI: 1.30–2.68, *P* < 0.001).Since these two groups differed on the basis of sociodemographic and risk profiles, we performed a multivariate logistic regression analysis adjusting for effects of age, sex, education, body mass index, history of smoking, and alcohol consumption. As indicated in Table 2, UBB participants were 68% more likely than their FBB counterparts to exhibit EDS. Results of each of the factors entered in the model are indicated in Table 2.Figure 1 illustrates the difference in EDS comparing UBB participants with FBB participants.

## 4. Discussion

Excessive daytime sleepiness is an important marker of insufficient nocturnal sleep, which results from a diagnosable sleep disorder or personal decision to reduce the amount of time spent in bed. There is ample evidence that individuals suffering from disorders such as insomnia, narcolepsy, or sleep apnea experience excessive daytime sleepiness [12]. Epidemiologic evidence demonstrates that the average sleep duration has declined from 8 hours, which was the norm 30 years ago, to 6.5 hours [13]. It is important to note that EDS has a considerable social and economic burden. Evidence shows that EDS leads to 100,000 car accidents in the United States annually [13, 14]. There is also evidence suggesting that the United States loses 16 billion dollars yearly due to loss of productivity, hours spent away from work, and medical conditions related to daytime sleepiness [15]. In light of these findings, public health advocates have been concerned that certain vulnerable populations might be at disproportionately worse levels of EDS-associated risk [16].

The main finding of our study is that UBB participants were nearly twice as likely as their FBB counterparts to exhibit EDS. This association remained significant even after adjusting for effects of sociodemographic and medical risk factors. To our knowledge, this is the first study that shows differences in EDS based on individuals' country of origin. Of note, levels of EDS observed for both groups were comparable to levels of EDS found in the Multiethnic Study of Atherosclerosis [17]. These data seem consistent with a previous community-based study we conducted [4]. In sum, these findings demonstrate further the importance of considering country of origin in the analysis of the epidemiologic sleep data. Thus, previous epidemiologic studies aggregating individuals from different countries of origin into one stratum (e.g., black, Hispanic, white, and others) may have been biased.

There are no data suggesting greater prevalence of sleep apnea among UBB compared with FBB. Since EDS is one of the main symptoms of sleep apnea, and because these patients have an existing diagnosis of hypertension, UBB may be at greater risk for sleep apnea, relative to their FBB counterparts. Unfortunately, the presence of and severity of sleep apnea was not assessed in this study. Hence, it is not clear why UBB have greater prevalence of EDS. If in fact it can be demonstrated that indeed UBB have greater prevalence of sleep apnea, public health efforts should be made to highlight the need for more research to understand why UBB are at greater risk for this condition. These findings also point to the need to examine the interrelationship among genetic [18], environmental [19, 20], and lifestyle [21] factors that might predispose one group to develop sleep apnea, more so than others.

Despite these findings there are important limitations to our study that should be noted. Our analyses were performed using subjective data, which do not always correspond well with objective data. Future studies should determine whether FBB and UBB will show differences in EDS measured objectively. Another important limitation of our study is that we did not have a control group. It would be important to assess the difference in the prevalence of EDS comparing hypertensive blacks with normotensive blacks.

## 5. Conclusions

Results of our study are important regarding evidence suggesting that individuals' country of origin should be considered in the analysis of epidemiologic sleep data in addition to their race and ethnicity. Specifically, we found that US-born hypertensive blacks are more likely to experience EDS compared with foreign-born hypertensive blacks.

## Figures and Tables

**Table 1:** Comparison of sociodemographic and health risk data of US-born black versus foreign-born black participating in the CAATCH.

Baseline characteristics based on participants' country of origin		
Variable	UBB (73%)	FBB (27%)
Age (mean ± SD)	56 ± 13	58 ± 13
Female gender (%)	71	73
Employed (%)	29	45*
High school (%)	31	23*
Alcohol consumption (%)	39	18*
Smoking history (%)	63	24*
Body mass index (mean ± SD)	31 ± 5.74	34 ± 8.01

*P < 0.001. Mean ± SD and (%) is reported.

**Table 2:** Regression coefficients of the EDS measure on country of origin (US-Born Black versus Foreign-Born Black), sociodemographic, and risk factors.

Associations of country of origin, sociodemographic, and risk factors with excessive daytime sleepiness			
Variable	OR	95% CI	*P*
US-Born Black	1.679	1.103–2.555	0.02
Age	0.989	0.975–1.004	0.15
Sex	0.857	0.598–1.227	0.40
Education	0.908	0.786–1.049	0.19
Employed	1.038	0.718–1.501	0.84
Alcohol consumption	0.850	0.588–1.227	0.39
Smoking habit	1.033	0.723–1.477	0.86
Body mass index	1.000	1.000-1.000	0.53
